# Anthropometric Indicators and Their Relationship with Physical Activity and Enjoyment in Childhood

**DOI:** 10.3390/jfmk11020168

**Published:** 2026-04-23

**Authors:** Aday Infante-Guedes, María del Carmen Carcelén-Fraile, Paulino Vico-Rodríguez, Marta Cano-Orihuela

**Affiliations:** 1 Department of Health Sciences, Faculty of Health Sciences, University of Atlántico Medio, 35017 Las Palmas de Gran Canaria, Spain; aday.infante@pdi.atlanticomedio.es (A.I.-G.);; 2 Department of Educational Sciences, Faculty of Social Sciences, University of Atlántico Medio, 35017 Las Palmas de Gran Canaria, Spain; 3 International Scientific Association on Innovation in Education and Health (ACIINES), 23007 Jaén, Spain; 4 International Network of Educational Law (RIIDE), 35017 Las Palmas de Gran Canaria, Spain

**Keywords:** physical fitness, adiposity, body composition, skinfold thickness, children

## Abstract

**Background:** Childhood is a key period for the development of body composition and physical activity habits that may influence health throughout life. Although physical activity has been widely associated with adiposity indicators, the role of enjoyment of physical activity as a motivational and affective component remains less explored. Therefore, the aim of this study was to analyze the relationship between several anthropometric indicators and both the level of physical activity and enjoyment of physical activity in schoolchildren. **Methods:** An observational, analytical, cross-sectional study was conducted with 386 schoolchildren (176 boys and 210 girls) with a mean age of 11.15 ± 0.66 years. Anthropometric indicators included body mass index, waist circumference, hip circumference, waist-to-hip ratio, and triceps and subscapular skinfold thickness. Physical activity level was assessed using the Physical Activity Questionnaire for Children (PAQ-C), and enjoyment of physical activity was evaluated using the Physical Activity Enjoyment Scale (PACES). Multiple linear regression analyses were performed, adjusting for age and sex. **Results:** Higher levels of physical activity were significantly associated with lower body mass index (B = −1.592; *p* < 0.001), waist circumference (B = −8.010; *p* < 0.001), hip circumference (B = −8.227; *p* < 0.001), waist-to-hip ratio (B = −0.008; *p* < 0.001), triceps skinfold thickness (B = −0.910; *p* = 0.002), and subscapular skinfold thickness (*p* < 0.05). Greater enjoyment of physical activity was significantly associated with lower body mass index (B = −1.778; *p* < 0.001), reduced waist circumference (B = −8.944; *p* < 0.001), hip circumference (B = −9.185; *p* < 0.001), waist-to-hip ratio (B = −0.008; *p* < 0.001), and triceps skinfold thickness (B = −1.100; *p* = 0.001). Greater enjoyment was also associated with lower anthropometric indicators of central adiposity (waist circumference and waist-to-hip ratio), whereas no significant association was observed with subscapular skinfold thickness (*p* = 0.066). **Conclusions:** Physical activity level and enjoyment of physical activity were associated with multiple anthropometric indicators in children, although physical activity showed more consistent associations, whereas enjoyment demonstrated a more selective pattern depending on the specific adiposity measure. These findings highlight the importance of considering both behavioral and affective dimensions of physical activity when promoting healthy morphofunctional development during childhood.

## 1. Introduction

Childhood is a critical stage for human morphological, functional, and behavioral development [1]. During this period, significant changes occur in body composition, musculoskeletal growth, and the acquisition of motor patterns, while physical activity habits are consolidated that can be maintained throughout life [2,3]. In this context, the study of factors associated with childhood body composition is fundamental to understanding the mechanisms that influence health and functional development from an early age.

Body composition in childhood is influenced by a complex interplay of biological, behavioral, and environmental factors [4]. Biological factors such as genetic predisposition and maturational status play a key role in determining growth patterns and fat distribution [5]. At the behavioral level, physical activity, sedentary behavior, and dietary habits are considered major modifiable determinants of adiposity. In addition, psychosocial factors, including motivation, self-perception, and enjoyment of physical activity, may influence children’s engagement in active behaviors and, consequently, their body composition [6]. Understanding how these different factors interact is essential for identifying early determinants of health and for designing effective interventions aimed at promoting healthy development during childhood [7].

From a functional perspective, different skinfold thicknesses may have distinct implications for motor behavior and physical performance [8]. Peripheral adiposity, commonly assessed through triceps skinfold thickness, may directly influence limb movement by increasing mechanical load and potentially reducing movement efficiency during dynamic tasks [9]. In contrast, central adiposity, estimated through subscapular skinfold thickness, may have a greater impact on trunk stability, postural control, and overall movement coordination [10]. These differences suggest that not all adiposity indicators affect physical function in the same way, highlighting the importance of analyzing multiple anthropometric measures when examining their relationship with physical activity and motor behavior in childhood [11].

Physical activity has been widely recognized as a key determinant of children’s health, with beneficial effects on body composition [12], cardiorespiratory function [13], muscle strength [14], and psychological well-being [15]. Several studies have shown that higher levels of physical activity are associated with a lower body mass index (BMI) and a reduced risk of excess adiposity in children and adolescents [16]. However, BMI has significant limitations, as it does not distinguish between fat mass and lean mass and does not adequately reflect the regional distribution of body fat, especially during periods of growth and development [17].

For this reason, the use of complementary anthropometric indicators, such as skinfold thickness and body circumference measurements, is considered essential for a more accurate assessment of adiposity and its distribution [18]. Waist circumference and waist-to-hip ratio allow for the estimation of central fat accumulation [19], while skinfold thickness provides specific information on peripheral and central subcutaneous adiposity [20]. From a morphofunctional perspective, these offer a more complete view of the child’s body profile and its potential relationship to motor behavior and physical function [21].

Despite abundant evidence on the relationship between physical activity and adiposity, most studies have focused exclusively on the amount of physical activity performed [22], paying less attention to the affective and motivational components of active behavior. Enjoyment of physical activity represents a relevant psychosocial dimension, especially in childhood, as it can decisively influence voluntary participation, long-term adherence, and the repetition of physical experiences [23]. Children who enjoy physical activity tend to engage more frequently and consistently in games and active activities, which could have significant implications for their physical and functional development [24]. From a motivational perspective, enjoyment of physical activity can be understood within the framework of Self-Determination Theory, which distinguishes between intrinsic and extrinsic forms of motivation [25]. According to this theory, intrinsic motivation, engaging in an activity for the inherent satisfaction it provides, is a key determinant of sustained behavioral engagement [26]. Enjoyment represents a central component of intrinsic motivation, reflecting a positive affective response to movement experiences. In children, higher levels of enjoyment are associated with greater voluntary participation, persistence, and long-term adherence to physical activity. Therefore, enjoyment may act as an important psychological mechanism linking behavioral engagement in physical activity with favorable morphofunctional outcomes [27]. However, evidence on the relationship between enjoyment of physical activity and anthropometric indicators in children is still limited and inconclusive. While some studies suggest that enjoyment may be associated with higher levels of physical activity and a healthier body profile [28,29], other studies have not found clear associations, especially when analyzing specific indicators of fat distribution [30,31]. Most previous studies have primarily focused on the quantity of physical activity and have often relied on general indicators such as body mass index, without considering more detailed measures of adiposity distribution. Furthermore, few studies have simultaneously examined the relationship between physical activity and enjoyment with multiple anthropometric indicators, including measures of global, peripheral, and central adiposity, making it difficult to understand whether these dimensions influence body composition similarly or differently and limiting a comprehensive understanding of their differential contributions to children’s morphofunctional development [32].

From a kinesiological perspective, it is important to distinguish between the amount of movement performed and the subjective experience associated with that movement. Although both dimensions are related, they are not equivalent and may contribute differently to morphological adaptations during childhood [33]. Analyzing physical activity and enjoyment together allows us to move toward a more comprehensive understanding of motor behavior and its relationship to physical health, moving beyond approaches focused solely on the volume of activity.

Therefore, the objective of this study was to analyze the relationship between various anthropometric indicators, including body mass index, skinfold thickness, and fat distribution indicators, with the level of physical activity and enjoyment of physical activity in children. Specifically, it aimed to examine whether physical activity and enjoyment are associated similarly or differently with global, peripheral, and central adiposity, adjusting for relevant sociodemographic variables such as age and sex. Understanding these relationships can provide valuable information for designing strategies to promote physical activity in childhood, highlighting not only the importance of increasing movement levels but also of fostering positive motor experiences that contribute to morphofunctional development and the adoption of active and healthy lifestyles from an early age. It was hypothesized that higher levels of physical activity would be associated with more favorable anthropometric profiles across multiple indicators, whereas enjoyment of physical activity would show more specific associations, particularly with body mass index and peripheral adiposity.

## 2. Materials and Methods

### 2.1. Study Design

This study followed an observational, analytical, cross-sectional design. It aimed to examine the associations between physical activity level, enjoyment of physical activity, and various anthropometric indicators in schoolchildren.

### 2.2. Participants

**Figure 1:**
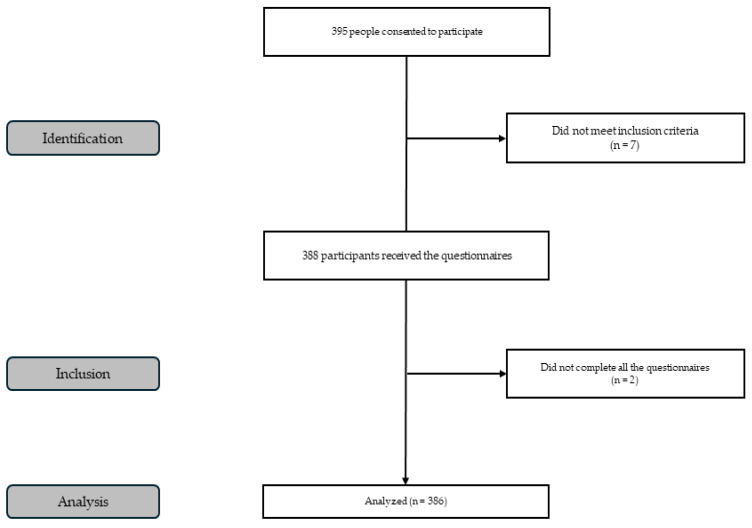
Flowchart of participants.

An observational, analytical, cross-sectional study was conducted with 386 schoolchildren, of whom 176 were boys (45.6%) and 210 were girls (54.4%). Initially, a total of 395 participants were contacted, resulting in a response rate of 97.72%. Participants were recruited using a non-probabilistic convenience sampling approach from primary schools that agreed to participate in the study. Inclusion criteria were: (i) being enrolled in primary education, (ii) being aged between 9 and 12 years, and (iii) having written informed consent provided by parents or legal guardians. Exclusion criteria included: (i) the presence of medical conditions or physical limitations that could interfere with participation in physical activity, and (ii) incomplete or missing data in the main study variables (Figure 1).

A sample size calculation was performed using the G*Power program (version 3.1) for a bivariate correlation analysis in a cross-sectional study. A two-tailed test, a significance level of α = 0.05, and a statistical power of 80% were assumed. Considering a small expected effect size (r = 0.15), a minimum sample size of 340 participants was estimated. A potential 10% loss due to incomplete questionnaires or invalid data was also considered, so the final required sample size was adjusted to 374 participants.

### 2.3. Instruments

#### 2.3.1. Dependent Variables

Anthropometric indicators were considered dependent variables in this study, as they represent physical characteristics that can influence children’s physical activity behaviors and related enjoyment. These included general body size measurements (height and weight, measured using an ASIMED^®^ Type B Class III digital scale (Barcelona, Spain) and a SECA^®^ 214 portable stadiometer (SECA Ltd., Hamburg, Germany), indicators of total adiposity (body mass index and skinfold thickness measured using a Slim Guide skinfold caliper (Creative Health Products, USA), and indicators of fat distribution (waist and hip circumferences and waist-to-hip ratio). Body mass index was calculated as weight in kilograms divided by height in meters squared. The triceps skinfold was considered an indicator of peripheral adiposity, whereas the subscapular skinfold was used as an indicator of central adiposity. Waist and hip circumferences were measured to assess central fat distribution, and the waist-to-hip ratio was calculated accordingly. All anthropometric measurements were performed by a single trained evaluator following standardized procedures. Although the technical error of measurement (TEM) was not formally calculated, several steps were taken to minimize measurement error, including the use of standardized protocols and duplicate measurements at each anatomical site. Two measurements were obtained for each variable, and the mean value was used for subsequent analyses. When the difference between the two measurements exceeded acceptable limits, a third measurement was performed, and the mean of the two closest values was used. These procedures are consistent with established anthropometric guidelines [34].

#### 2.3.2. Independent Variables

The level of usual physical activity was assessed using the Physical Activity Questionnaire for Children (PAQ-C) [35,36]. This instrument is a self-administered questionnaire designed to estimate moderate to vigorous physical activity performed during the past 7 days in children (approximately 8 to 14 years old). The PAQ-C consists of 9 items scored on a 5-point Likert scale, where 1 indicates a very low level of physical activity and 5 a very high level. The items assess physical activity performed in different contexts, including free time and leisure, physical education classes, recess, after school, weekends and an overall assessment of weekly activity level. Item 10 is descriptive and is used to identify possible atypical circumstances (e.g., illness or injury), so it is not included in the calculation of the total score. The overall PAQ-C score is obtained by calculating the arithmetic mean of the 9 scored items, resulting in a continuous value between 1 and 5, where higher values indicate greater levels of physical activity. The questionnaire does not establish clinical cut-off points, so the results are interpreted continuously or comparatively between groups. The PAQ-C showed acceptable reliability (Cronbach’s alpha = 0.745) in the present sample.

Enjoyment of physical activity was assessed using the Physical Activity Enjoyment Scale (PACES), in its adapted version by Molt [37,38]. This is a structured self-report questionnaire designed to measure the degree of enjoyment experienced during physical activity. The instrument consists of 16 items with responses on a 5-point Likert scale, ranging from 1 (“strongly disagree”) to 5 (“strongly agree”). The questionnaire includes statements phrased positively and negatively regarding the affective experience associated with physical activity. The total score is obtained from the arithmetic mean of the 16 items, after recoding the negatively worded items, resulting in a value between 1 and 5, where higher scores indicate a greater level of enjoyment of physical activity. Although the PACES assesses enjoyment as a global construct, some studies have considered the presence of two dimensions (positive affect and negative affect). However, in the present study a total score was used, in accordance with its most common use in child and adolescent populations [39]. The PACES demonstrated good internal consistency (Cronbach’s alpha = 0.801) in the present sample.

#### 2.3.3. Confounding Variables

Age and sex were included as primary confounding variables, given their well-established influence on both anthropometric characteristics and physical activity behaviors in childhood [40]. These variables were included as covariates in all adjusted analyses to control for their potential confounding effects. Sociodemographic data were collected using a structured questionnaire that included information on participants’ age and family background.

### 2.4. Procedures

The study was first presented to the school management teams, and institutional authorization was obtained prior to data collection. Subsequently, informed consent was obtained from parents or legal guardians of all participants. Each participant was assigned a unique anonymous code to ensure confidentiality. Data collection was carried out during school hours in scheduled sessions organized in collaboration with the teaching staff. All measurements were conducted in the morning (between 9:00 and 11:00 h) to minimize potential variability related to daily fluctuations in anthropometric measures. Anthropometric measurements were conducted by the same evaluator following standardized protocols. Measurements were taken in a designated space within the school under controlled conditions to ensure accuracy and consistency. The questionnaires assessing physical activity (PAQ-C) and enjoyment of physical activity (PACES) were administered collectively in the classroom setting. Participants received standardized instructions, and the research team was present at all times to clarify any doubts and ensure proper understanding of the items. Teachers were present during the assessment sessions to facilitate organization and ensure an appropriate environment. All procedures were conducted under standardized conditions to guarantee data quality and consistency.

### 2.5. Statistical Analysis

Statistical analyses were performed using SPSS (version 23). Descriptive statistics are presented as mean ± standard deviation for continuous variables and frequencies and percentages for categorical variables. Independent samples t-tests were used to assess sex differences in continuous variables and Chi-square tests were used for categorical variables. To examine the relationship between physical activity level (PAQ-C) and anthropometric and body composition variables (body mass index, waist circumference, hip circumference, and waist-to-hip ratio), multiple linear regression analyses were performed. Regression coefficients (B) represent the expected change in each anthropometric variable (expressed in their original units, e.g., centimeters or mm) associated with a one-unit increase in the independent variables (PAQ-C or PACES scores). Standardized regression coefficients (β) were also calculated to facilitate comparison of effect sizes across variables measured on different scales. Anthropometric variables were considered dependent variables, while physical activity level was the independent variable, with models adjusted for age and sex. Multiple linear regression was selected as an appropriate method to examine the association between continuous independent variables and multiple continuous dependent variables, allowing adjustment for potential confounders. Effect sizes were reported using regression coefficients (B) and adjusted coefficients of determination (adjusted R^2^) as the primary estimate of explained variance, and 95% confidence intervals were calculated to assess the precision of the estimates. Prior to analysis, the assumptions of linear regression were evaluated, including normality of residuals, homoscedasticity, and absence of multicollinearity. Multicollinearity among independent variables was additionally assessed using the variance inflation factor (VIF). These assumptions were considered acceptable based on standard diagnostic procedures. Given the cross-sectional design, the analyses were conducted to examine associations between variables, and no causal inferences can be established. Statistical significance was set at *p* < 0.05.

### 2.6. Ethical Considerations

The study was conducted in accordance with the Declaration of Helsinki, and approved by the Ethics Committee of Mild-Atlantic University (Approval Code: CEI/01-032).

## 3. Results

The mean age of the sample was 11.15 ± 0.66 years, with no significant differences between boys and girls (*p* = 0.278). Most participants were enrolled in fifth grade (61.7%), followed by sixth grade (27.2%), fourth grade (8.5%), and third grade (2.6%), with no significant differences by sex (*p* = 0.239). No statistically significant sex differences were observed in anthropometric variables, including height, weight, body mass index (BMI), triceps and subscapular skinfold thicknesses, waist and hip circumferences, or waist-to-hip ratio (all *p* > 0.05). Similarly, maternal education level did not differ significantly between boys and girls (*p* = 0.240). Regarding behavioral variables, no significant sex differences were found in physical activity enjoyment or in the level of physical activity assessed by the PAQ-C questionnaire (both *p* > 0.05). Detailed sociodemographic, anthropometric, and physical activity characteristics of the sample by sex are presented in Table 1.

### 3.1. Association Between Physical Activity and Anthropometric Indicators

#### 3.1.1. Body Mass Index

The association between physical activity level, assessed using the PAQ-C, and body mass index (BMI) is presented in Table 2, adjusted for age and sex. Age and sex were not significantly associated with BMI in the adjusted model (*p* > 0.05). Higher levels of physical activity were significantly associated with lower BMI values (B = −1.592; SE = 0.028; *β* = −0.946; 95% CI [−1.647, −1.537]; *p* < 0.001). The overall model was statistically significant and explained 1.94% of the variance in BMI (adjusted R^2^ = 0.194; F(3,382) = 1081.52, *p* < 0.001).

#### 3.1.2. Indicators of Fat Distribution

Table 3 presents the associations between physical activity level, assessed using the PAQ-C, and indicators of fat distribution, adjusted for age and sex. Higher levels of physical activity were significantly associated with lower waist circumference, hip circumference, and waist-to-hip ratio (all *p* < 0.001). Age and sex were not significantly associated with the anthropometric outcomes in the adjusted models (*p* > 0.05). Higher levels of physical activity were significantly associated with lower waist circumference, hip circumference, and waist-to-hip ratio. The regression models were statistically significant (F(3,382) = 1464.83, *p* < 0.001; F(3,382) = 3078.65, *p* < 0.001; and F(3,382) = 438.74, *p* < 0.001, respectively) and explained 16.2%, 16.0%, and 27.5% of the variance in waist circumference, hip circumference, and waist-to-hip ratio, respectively (adjusted R^2^ = 0.162, 0.160, and 0.275).

#### 3.1.3. Skinfold Thickness

Table 4 presents the associations between physical activity level, assessed using the Physical Activity Questionnaire for Children (PAQ-C), and skinfold thickness indicators (triceps and subscapular), adjusted for age and sex. Higher levels of physical activity were significantly associated with lower triceps skinfold thickness (*p* = 0.002). In this model, sex also showed a significant association with triceps skinfold thickness (*p* = 0.031), while age was not significantly associated (*p* > 0.05). Regarding subscapular skinfold thickness, physical activity level was inversely associated with this indicator (*p* = 0.024); however, neither age nor sex showed statistically significant associations in the adjusted model (*p* > 0.05). Higher levels of physical activity were significantly associated with lower triceps skinfold thickness and lower subscapular skinfold thickness. The triceps skinfold model was statistically significant (F(3,382) = 5.74, *p* = 0.001) and explained 3.6% of the variance (adjusted R^2^ = 0.036), whereas the subscapular skinfold model was not statistically significant (F(3,382) = 2.06, *p* = 0.105) and explained 0.8% of the variance (adjusted R^2^ = 0.008).

**Table 3:** Association between physical activity level (PAQ-C) and indicators of fat distribution, adjusted for age and sex.

Variable	Waist Circumference						Hip Circumference						Waist-to-Hip Ratio					
	B	SE	β	95% CI [lower–upper]	VIF	*p*	B	SE	β	95% CI [lower–upper]	VIF	*p*	B	SE	β	95% CI [lower–upper]	VIF	*p*
**Age (years)**	0.010	0.052	0.002	[−0.092, 0.113]	1.007	0.842	−0.010	0.055	−0.002	[−0.118, 0.097]	1.007	0.852	0.000	0.000	0.048	[0.000, 0.001]	1.007	0.051
**Sex**	−0.038	0.069	−0.006	[−0.175, 0.098]	1.014	0.581	−0.044	0.073	−0.006	[−0.187, 0.099]	1.014	0.546	0.000	0.000	0.003	[]	1.014	0.907
**Physical activity level (PAQ-C)**	−8.010	0.082	−0.981	[−8.171, −7.849]	1.008	<0.001	−8.227	0.086	−0.980	[−8.396, −8.058]	1.008	<0.001	−0.008	0.000	−0.878	[−0.008, −0.007]	1.008	<0.001
	Adjusted R^2^	0.162					Adjusted R^2^	0.160					Adjusted R^2^	0.275				
	F(3,382) = 1464.834, *p* < 0.001						F(3,382) = 3078.649, *p* < 0.001						F(3,382) = 438.736, *p* < 0.001					

Note: Non-standardized regression coefficient (B), standard error (SE), standardized regression coefficient (β), CI = confidence interval, PAQ-C = the Physical Activity Questionnaire for Children. Sex was coded as 0 = boy (reference category) and 1 = girl.

**Table 4:** Association between physical activity level (PAQ-C) and indicators of skinfold thickness, adjusted for age and sex.

Variable	Triceps Skinfold						Subscapular Skinfold					
	B	SE	β	95% CI [lower–upper]	VIF	*p*	B	SE	β	95% CI [lower–upper]	VIF	*p*
Age (years)	0.223	0.183	0.061	[−0.137, 0.583]	1.007	0.225	0.064	0.104	0.031	[−0.141, 0.268]	1.007	0.581
Sex	0.528	0.244	0.109	[0.049, 1.007]	1.014	0.031	0.192	0.138	0.071	[−0.081,0.464]	1.014	0.186
Physical activity level (PAQ-C)	−0.910	0.287	−0.159	[−1.474, −0.345]	1.008	0.002	−0.305	0.163	−0.095	[−0.625, 0.016]	1.008	0.024
	Adjusted R^2^	0.036					Adjusted R^2^	0.008				
	F(3,382) = 5.738, *p* = 0.001						F(3,382) = 2.058, *p* = 0.105					

Note: Non-standardized regression coefficient (B), standard error (SE), standardized regression coefficient (β), CI = confidence interval, PAQ-C = the Physical Activity Questionnaire for Children. Sex was coded as 0 = boy (reference category) and 1 = girl.

### 3.2. Association Between Enjoyment of Physical Activity and Anthropometric Indicators

#### 3.2.1. Body Mass Index

Table 5 presents the associations between enjoyment of physical activity, assessed using the Physical Activity Enjoyment Scale (PACES), and body mass index (BMI), adjusted for age and sex. The overall model was statistically significant (*F(3,382) = 930.24, p < 0.001*) and explained 28.0% of the variance in BMI (adjusted R^2^ = 0.280). Neither age nor sex were significantly associated with BMI in the adjusted model (*p* > 0.05). Higher levels of enjoyment of physical activity were significantly associated with lower BMI values. 

#### 3.2.2. Indicators of Fat Distribution

Table 6 presents the associations between enjoyment of physical activity, assessed using the Physical Activity Enjoyment Scale (PACES), and indicators of fat distribution (waist circumference, hip circumference, and waist-to-hip ratio), adjusted for age and sex. Age and sex were not significantly associated with any of the anthropometric outcomes in the adjusted models, except for age, which showed a small but significant association with waist-to-hip ratio (*p* = 0.045). Higher levels of enjoyment of physical activity were significantly associated with lower waist circumference, hip circumference, and waist-to-hip ratio. The models were statistically significant (F(3,382) = 218.70, *p* < 0.001; F(3,382) = 139.93, *p* < 0.001; and F(3,382) = 13.11, *p* < 0.001, respectively) and explained 14.6%, 14.4%, and 27.4% of the variance in waist circumference, hip circumference, and waist-to-hip ratio, respectively (adjusted R^2^ = 0.146, 0.144, and 0.274).

#### 3.2.3. Skinfold Thickness

Table 7 presents the associations between physical activity enjoyment, assessed using the Physical Activity Enjoyment Scale (PACES), and skinfold thickness (triceps and subscapular), adjusted for age and sex. Higher levels of enjoyment of physical activity were significantly associated with lower triceps skinfold thickness. In contrast, no statistically significant association was observed between enjoyment of physical activity and subscapular skinfold thickness. Age was not significantly associated with either skinfold, while sex showed a significant association with triceps skinfold thickness but not with subscapular skinfold. The triceps skinfold model was statistically significant (F(3,382) = 6.27, *p* < 0.001) and explained 3.9% of the variance (adjusted R^2^ = 0.039), whereas the subscapular skinfold model was not statistically significant (F(3,382) = 2.03, *p* = 0.109) and explained 0.8% of the variance (adjusted R^2^ = 0.008).

## 4. Discussion

The aim of this study was to analyze the relationship between various anthropometric indicators and two key dimensions of active behavior in childhood: the level of physical activity and the enjoyment of physical activity. The main findings indicate that physical activity is consistently associated with most of the anthropometric indicators analyzed, including body mass index, peripheral adiposity, and indicators of fat distribution. In contrast, the enjoyment of physical activity showed a more specific pattern of associations. It was primarily with BMI and triceps skinfold thickness and was also significantly associated with anthropometric indicators of fat distribution such as waist circumference and waist-to-hip ratio. However, no significant association was observed with subscapular skinfold thickness, a skinfold-based indicator of central adiposity. These results suggest that the amount of physical activity and the affective experience associated with movement contribute differently to the anthropometric profile in childhood.

Regarding physical activity, our results show significant inverse associations with BMI, waist circumference, hip circumference, waist-to-hip ratio, and triceps skinfold thickness. These findings are consistent with previous literature that identifies regular physical activity as a key determinant of energy balance and body fat accumulation during childhood [41]. The consistency of these associations across different anthropometric indicators reinforces the idea that BMI, while useful, does not by itself reflect changes in the distribution and location of body fat [42]. From a morphofunctional perspective, regular physical activity is associated with greater muscle activation and energy expenditure and adaptations in the musculoskeletal system that can limit the accumulation of adipose tissue [43]. The observed association with waist circumference and waist-to-hip ratio is particularly relevant, given that these indicators reflect central fat distribution, which has been linked to a higher cardiometabolic risk, even at young ages. A more favorable fat distribution profile could be associated with functional aspects such as trunk stability or movement efficiency; however, these variables were not directly assessed in the present study and should be interpreted with caution [44].

Although some unstandardized regression coefficients appear large, particularly for anthropometric variables expressed in centimeters (e.g., waist and hip circumference), these values reflect the change associated with a one-unit increase in PAQ-C or PACES scores. Given that these scales range from 1 to 5, a one-unit increase represents a meaningful behavioral difference rather than a minimal variation. Therefore, the magnitude of these coefficients should be interpreted in the context of the measurement scales. The inclusion of standardized coefficients (β) further supports the interpretation of effect sizes and allows comparison across variables.

Analysis of skinfold thicknesses showed that physical activity is significantly associated with the triceps skinfold, but not with the subscapular skinfold. This differential pattern suggests that peripheral adiposity may be more sensitive to the mechanical and metabolic stimuli derived from habitual physical activity [9]. The triceps skinfold, located in a region actively involved in movement and play, may more accurately reflect the cumulative effects of daily physical activity [45]. Conversely, the lack of association with the subscapular skinfold indicates that subcutaneous fat located in the trunk may be more influenced by biological factors, such as maturational stage, hormonal regulation, or genetic predisposition. In this sense, physical activity alone may not be sufficient to modify certain central fat deposits during childhood, especially in cross-sectional studies.

One of the most relevant aspects of this study is the inclusion of enjoyment of physical activity as an independent variable. Our results show that greater enjoyment is significantly associated with lower BMI values and reduced triceps skinfold thickness, even after adjusting for age and sex. These findings suggest that the affective and motivational components of physical activity may play an important role in children’s body composition. From a behavioral and kinesiological perspective, enjoyment can facilitate active behavior, promoting more frequent, voluntary, and sustained participation in physical activities [46]. Children who enjoy physical activity tend to engage in a wider variety of movements, games, and motor tasks, which may contribute to greater cumulative energy expenditure and potentially more favorable morphological adaptations, especially peripherally [47]. However, while enjoyment was significantly associated with anthropometric indicators of central adiposity, such as waist circumference and waist-to-hip ratio, it did not show a significant association with subscapular skinfold thickness. This distinction is important, as it highlights that different indicators of central adiposity may capture distinct physiological aspects. Anthropometric measures such as waist circumference reflect overall fat distribution, whereas skinfold thickness provides a more localized estimate of subcutaneous fat. Therefore, the relationship between enjoyment and adiposity may vary depending on the specific measurement used [48]. This result indicates that, although enjoyment can influence global and peripheral adiposity, its association with central adiposity appears to depend on the specific indicator considered, showing consistent relationships with anthropometric measures (waist circumference and waist-to-hip ratio) but not with subscapular skinfold thickness. It is also important to consider the potential bidirectional nature of this relationship. While enjoyment of physical activity may promote greater engagement and influence body composition, it is also possible that children with more favorable anthropometric profiles may experience physical activity as more enjoyable. This reciprocal relationship cannot be disentangled within the cross-sectional design of the present study and should be explored in future longitudinal research.

Taken together, this study’s results highlight that physical activity and enjoyment represent related but not equivalent dimensions of motor behavior. While the level of physical activity showed broad and consistent associations with most anthropometric indicators, enjoyment exhibited a more selective pattern. This distinction is relevant from a functional perspective, as it suggests that increasing the amount of movement is key to influencing central adiposity, while fostering positive experiences during physical activity could be especially important for promoting changes in peripheral adiposity and BMI. These findings support the need to address children’s physical activity from a multidimensional perspective, considering both the quantity and quality of the motor experience [48]. Ignoring the affective dimension could limit the effectiveness of interventions aimed at improving body composition and long-term health.

### 4.1. Practical Implications

From an applied perspective, the results suggest that programs aimed at improving body composition in childhood should combine strategies focused on increasing physical activity levels with interventions that promote enjoyment of movement. In the school setting, physical education classes and extracurricular activities should be designed to be engaging, varied, and adapted to children’s developmental level; while still ensuring they achieve sufficient activity levels. Fostering enjoyment of physical activity can contribute to improved long-term adherence, facilitating the development of active habits that are maintained throughout adolescence and adulthood. This approach can have benefits not only on body composition but also on motor skills, self-esteem, and overall well-being.

### 4.2. Limitations and Strengths

This study has several limitations that should be considered. Its cross-sectional design prevents establishing causal relationships between physical activity, enjoyment, and anthropometric indicators. Furthermore, physical activity and enjoyment were assessed using self-reported questionnaires, which are subject to recall bias and social desirability bias. These limitations may lead to overestimation or underestimation of actual behaviors and perceptions. In addition, self-reported measures may not accurately capture the intensity, frequency, or context of physical activity compared to objective methods such as accelerometry. Therefore, this limitation may affect the precision of the observed associations and should be considered when interpreting the results. Additionally, important potential confounding variables such as biological maturation, dietary habits, and socioeconomic status were not included in the analyses. These factors are known to influence body composition and fat distribution during childhood and may also be associated with levels of physical activity and enjoyment. Their omission may have introduced residual confounding, potentially affecting the magnitude and direction of the observed associations. Therefore, the findings should be interpreted with caution, and future studies should incorporate these variables to provide a more comprehensive understanding of the determinants of anthropometric outcomes in children. However, the study also has significant strengths. The inclusion of multiple anthropometric indicators allows for a more comprehensive assessment of adiposity, overcoming the limitations of relying solely on BMI. Moreover, the simultaneous analysis of physical activity and enjoyment provides an integrative perspective that combines behavioral and affective dimensions of movement, contributing to a better understanding of the factors associated with body composition in childhood.

## 5. Conclusions

The aim of this study was to examine the association between physical activity level, enjoyment of physical activity, and multiple anthropometric indicators in children. The findings indicate that physical activity level was consistently associated with several anthropometric outcomes, showing moderate associations with indicators such as body mass index and fat distribution (R^2^ ≈ 0.12–0.27), and smaller associations with skinfold thickness (R^2^ ≈ 0.01–0.04). Enjoyment of physical activity was also associated with selected anthropometric indicators, including body mass index, triceps skinfold thickness, and anthropometric measures of fat distribution (waist circumference and waist-to-hip ratio), but not with subscapular skinfold thickness. These results highlight the importance of distinguishing between different indicators of adiposity, as associations may vary depending on the measurement method used. Overall, these findings suggest that physical activity and enjoyment represent related but distinct dimensions of behavior, each showing different patterns of association with anthropometric outcomes. Given the cross-sectional design, these results should be interpreted as associations rather than causal relationships.

## Figures and Tables

**Table 1:** Sociodemographic, anthropometric, and physical activity-related characteristics of the sample by sex.

VARIABLE		TOTAL (N = 386)	BOYS (N = 176)	GIRLS (N = 210)	*p* VALUE
**AGE (YEARS)**		11.15 ± 0.66	11.20 ± 0.05	11.10 ± 0.04	0.278
**GRADE (%)**	3º	10 (2.60%)	3 (0.80%)	7 (1.80%)	0.239
	4º	33 (8.50%)	15 (3.90%)	18 (4.70%)	
	5º	238 (61.70%)	104 (26.90%)	134 (37.40%)	
	6º	105 (27.20%)	54 (14.0%)	51 (13.20%)	
**HEIGHT (CM)**		151 ± 0.06	151 ± 0.04	152 ± 0.03	0.860
**WEIGHT (KG)**		45.04 ± 4.95	44.61 ± 0.37	45.40 ± 0.34	0.895
**BMI (KG/M2)**		19.65 ± 0.71	19.59 ± 0.05	19.70 ± 0.05	0.521
**MATERNAL EDUCATION LEVEL (%)**	No studies	44 (11.40%)	13 (3.40%)	31 (8.00%)	0.240
	Primary studies	56 (14.50%)	28 (7.30%)	28 (7.30%)	
	Secondary studies	153 (39.60%)	74 (19.20%)	79 (20.50%)	
	University studies	133 (34.50%)	61 (15.80%)	72 (18.70%)	
**TRICEPS SKINFOLD THICKNESS (MM)**		14.95 ± 1.99	14.78 ± 0.15	15.10 ± 0.14	0.927
**SUBSCAPULAR SKINFOLD THICKNESS (MM)**		9.86 ± 1.41	9.75 ± 0.10	9.96 ± 0.09	0.899
**WAIST CIRCUMFERENCE** **(CM)**		66.37 ± 3.44	66.08 ± 0.26	66.61 ± 0.24	0.801
**HIP CIRCUMFERENCE (CM)**		73.22 ± 3.54	72.92 ± 0.27	73.47 ± 0.24	0.837
**WAIST-TO-HIP RATIO**		0.91 ± 0.03	0.90 ± 0.03	0.91 ± 0.02	0.303
**PHYSICAL ACTIVITY ENJOYMENT (SCORE)**		3.35 ± 0.42	3.31 ± 0.31	3.38 ± 0.29	0.759
**LEVEL OF PHYSICAL ACTIVITY (PAQ-C SCORE)**		4.04 ± 0.37	4.01 ± 0.28	4.07 ± 0.26	0.708

**Table 2:** Association between physical activity level (PAQ-C) and body mass index (BMI), adjusted for age and sex.

Variable	BMI					
	B	SE	β	95% CI [lower–upper]	VIF	*p*
Age (years)	0.006	0.018	0.006	[−0.029, 0.042]	1.007	0.718
Sex	−0.006	0.024	−0.004	[−0.053, 0.041]	1.014	0.806
Physical activity level (PAQ-C)	−1.592	0.028	−0.946	[−1.647, −1.537]	1.008	<0.001
Adjusted R^2^	0.194					
**F(3,382) = 1081.516, *p* < 0.001**						

Note: Non-standardized regression coefficient (B), standard error (SE), standardized regression coefficient (β), CI = confidence interval, BMI = body mass index (kg/m^2^). Sex was coded as 0 = boy (reference category) and 1 = girl.

**Table 5:** Association between enjoyment of physical activity and body mass index, adjusted for age and sex.

Variable	BMI					
	B	SE	β	95% CI [lower–upper]	VIF	*p*
Age (years)	0.009	0.019	0.009	[−0.028, 0.047]	1.007	0.627
Sex	−0.005	0.025	−0.004	[−0.055, 0.045]	1.014	0.838
PACES score	−1.778	0.034	−0.938	[−1.844, −1.711]	1.008	<0.001
Adjusted R^2^	0.280					
**F(3,382) = **930.237**, *p* < 0.001**						

Note: Non-standardized regression coefficient (B), standard error (SE), standardized regression coefficient (β), CI = confidence interval, PACES = Physical Activity Enjoyment Scale. Sex was coded as 0 = boy (reference category) and 1 = girl.

**Table 6:** Association between enjoyment of physical activity (PACES) and indicators of fat distribution, adjusted for age and sex.

Variable	Waist Circumference						Hip Circumference						Waist-to-Hip Ratio					
	B	SE	β	95% CI [lower–upper]	VIF	*p*	B	SE	β	95% CI [lower–upper]	VIF	*p*	B	SE	β	95% CI [lower–upper]	VIF	*p*
**Age (years)**	0.025	0.062	0.005	[−0.098, 0.147]	1.007	0.693	0.004	0.065	0.001	[−0.124, 0.132]	1.007	0.946	0.000	0.000	0.050	[0.000, 0.001]	1.007	0.045
**Sex**	−0.035	0.083	−0.005	[−0.198, 0.128]	1.014	0.671	−0.041	0.087	−0.006	[−0.211, 0.130]	1.014	0.639	0.000	0.000	0.003	[0.000, 0.000]	1.014	0.900
**PACES score**	−8.944	0.110	−0.973	[−9.160, −8.728]	1.008	<0.001	−9.185	0.115	−0.972	[−9.412, −8.959]	1.008	<0.001	−0.008	0.000	−0.872	[−0.009, −0.008]	1.008	<0.001
	Adjusted R^2^	0.146					Adjusted R^2^	0.144					Adjusted R^2^	0.274				
	F(3,382) = 218.696, *p* < 0.001						F(3,382) = 139.929, *p* < 0.001						F(3,382) = 13.114, *p* < 0.001					

Note: Non-standardized beta regression coefficient (B), standard error (SE), standardized regression coefficient (β), CI = confidence interval, PACES = Physical Activity Enjoyment Scale. Sex was coded as 0 = boy (reference category) and 1 = girl.

**Table 7:** Association between physical activity enjoyment (PACES) and skinfold thickness, adjusted for age and sex.

Variable	Triceps Skinfold						Subscapular Skinfold					
	B	SE	β	95% CI [lower–upper]	VIF	*p*	B	SE	β	95% CI [lower–upper]	VIF	*p*
**Age (years)**	0.223	0.183	0.061	[−0.136, 0.583]	1.007	0.222	0.064	0.104	0.031	[−0.140, 0.269]	1.007	0.537
**Sex**	0.523	0.243	0.108	[0.044, 1.001]	1.014	0.032	0.192	0.138	0.071	[−0.080, 0.464]	1.014	0.167
**PACES score**	−1.100	0.323	−0.171	[−1.735, −0.465]	1.008	0.001	−0.339	0.184	−0.094	[−0.700, 0.022]	1.008	0.066
	Adjusted R^2^	0.039					Adjusted R^2^	0.008				
	F(3,382) = 6.274, *p* < 0.001						F(3,382) = 2.031, *p* = 0.109					

Note: Non-standardized beta regression coefficient (B), standard error (SE), standardized regression coefficient (β), CI = confidence interval, PACES = Physical Activity Enjoyment Scale. Sex was coded as 0 = boy (reference category) and 1 = girl.

## Data Availability

The data presented in this study are available upon request from the corresponding author. The data are not publicly available because, due to the sensitive nature of the questions asked in this study, participants were assured raw data would remain confidential and would not be shared.

## Abbreviations

The following abbreviations are used in this manuscript:

BMI	Body Mass Index
PAQ-C	Physical Activity Questionnaire for Children
PACES	Physical Activity Enjoyment Scale
SE	Standard Error
